# Circulating Exosomal miR-221 from Maternal Obesity Inhibits Angiogenesis via Targeting *Angptl2*

**DOI:** 10.3390/ijms221910343

**Published:** 2021-09-26

**Authors:** Yuanfei Zhou, Mao Xia, Chenbin Cui, Hongkui Wei, Siwen Jiang, Jian Peng

**Affiliations:** 1 Department of Animal Nutrition and Feed Science, College of Animal Science and Technology, Huazhong Agricultural University, Wuhan 430070, China; zhouyuanfei@mail.hzau.edu.cn (Y.Z.); xiamao_1992@163.com (M.X.); cuichenbin@webmail.hzau.edu.cn (C.C.); weihongkui@mail.hzau.edu.cn (H.W.); 2 Key Laboratory of Swine Genetics and Breeding of Agricultural Ministry, Key Laboratory of Agricultural Animal Genetics, Breeding and Reproduction of Ministry of Education, College of Animal Science and Technology, Huazhong Agricultural University, Wuhan 430070, China; 3 The Cooperative Innovation Center for Sustainable Pig Production, Wuhan 430070, China

**Keywords:** maternal obesity, circulating exosome, angiogenesis, miR-221

## Abstract

Maternal obesity disrupts both placental angiogenesis and fetus development. However, the links between adipocytes and endothelial cells in maternal obesity are not fully understood. The aim of this study was to characterize exosome-enriched miRNA from obese sow’s adipose tissue and evaluate the effect on angiogenesis of endothelial cells. Plasma exosomes were isolated and analyzed by nanoparticle tracking analysis (NTA), electron morphological analysis, and protein marker expression. The number of exosomes was increased as the gestation of the sows progressed. In addition, we found that exosomes derived from obese sows inhibited endothelial cell migration and angiogenesis. miRNA detection showed that miR-221, one of the miRNAs, was significantly enriched in exosomes from obese sows. Further study demonstrated that exosomal miR-221 inhibited the proliferation and angiogenesis of endothelial cells through repressing the expression of *Angptl2* by targeting its 3′ untranslated region. In summary, miR-221 was a key component of the adipocyte-secreted exosomal vesicles that mediate angiogenesis. Our study may be a novel mechanism showing the secretion of “harmful” exosomes from obesity adipose tissues causes placental dysplasia during gestation.

## 1. Introduction

In modern society, obesity has become a serious hazard to human health. Maternal obesity impacts both the placenta and the fetus, often resulting in fetal overgrowth and a greater frequency of large for gestational age (LGA) infants [1]. However, incidences of small for gestational age (SGA) and low birth weight (LBW) in obese pregnancies have also been reported [2]. Similarly, in sows, excessive backfat of gestation increased the proportion of intrauterine growth restriction (IUGR) and resulted in lower litter weight gain [3,4]. The placental tissue plays a crucial role in substance exchange between the fetus and the mother during pregnancy [5]. The development of the placental vascular network directly affects the blood flow rate of the uterus and umbilical cord, which is the key to guarantee placental function and fetal development [6]. Studies have shown that maternal obesity promotes a lipotoxic placental environment and inhibits placental angiogenesis in humans [7], as well as in some animals, such as ewes [8] and sows [3]. This in turn causes placental morphology, blood flow, fetomaternal exchanges, and endocrine function [9]. Nevertheless, maternal obesity disrupts normal placental angiogenesis, and its impact on fetal growth has not been studied thoroughly.

Adipose tissue is an active endocrine organ, which plays an important role in the development of obesity and its related diseases [10]. Recently, adipocyte-derived exosomes have played key roles in cell-to-cell communication and conveying molecular signals to cells at proximal as well as distal locations [11,12]. Exosomes belong to the family of extracellular vesicles, with a typical size of 30–100 nm [13]. Exosomes bear various biological molecules including microRNAs (miRNAs), proteins, RNAs, and DNAs, which regulate the phenotype of target cells [11]. Studies have reported that adipose tissue is a major source of circulating miRNAs [13]. The obesity adipocyte-derived exosomal miR-27a induced insulin resistance in skeletal muscle through miR-27a-mediated repression of the peroxisome proliferator-activated receptor γ(PPARγ) [14]. Whether obesity-derived exosomal miRNAs regulate placental angiogenesis is unknown.

In this study, we hypothesized that exosomes derived from circulating could be enriched with miRNA that would induce the angiogenesis of endothelial cells. We revealed that obesity-derived exosomes could inhibit the angiogenesis of endothelial cells. In particular, miR-221, which was enriched in exosomes from obese sows, could inhibit angiogenesis by targeting angiopoietin-like protein 2 (Angpl2), a positive regulator of angiogenesis [15]. Therefore, this study expands our understanding of the effect of maternal body condition on placental development.

## 2. Results

### 2.1. Exosome Isolation and Characterization

**Figure 1:**
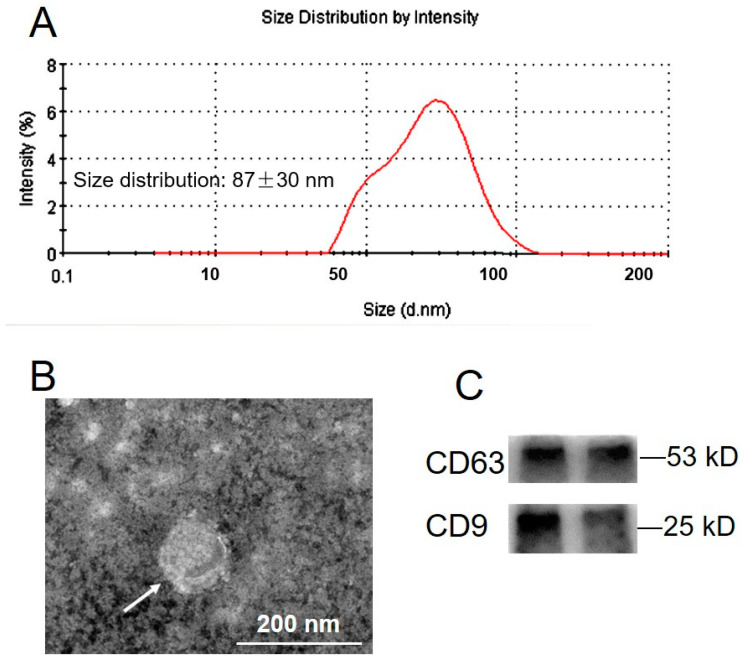
Isolation and characterization of exosomes derived from sow plasma. (**A**) Nanoparticle tracking analysis (NTA) of size distributions of exosomes. (**B**) Morphology of exosomes under transmission electron microscopy. (**C**) Western blotting was performed to detect the expression of exosomal markers CD63 and CD9 in plasma-derived exosomes.

Plasma exosomes were isolated using the standard methods [16]. Nanoparticle tracking analysis showed that particles ranged from 40 to 120 nm in diameter, with an average of 87 ± 30 nm (mean ± SD) (Figure 1A). Electron morphological analysis revealed the cup-shape characteristics of exosome vesicles (Figure 1B). CD63 and CD9 were detected as positive for exosomal markers (Figure 1C).

### 2.2. Exosome Concentration during Gestation Stage in Normal and Obese Sows

**Figure 2:**
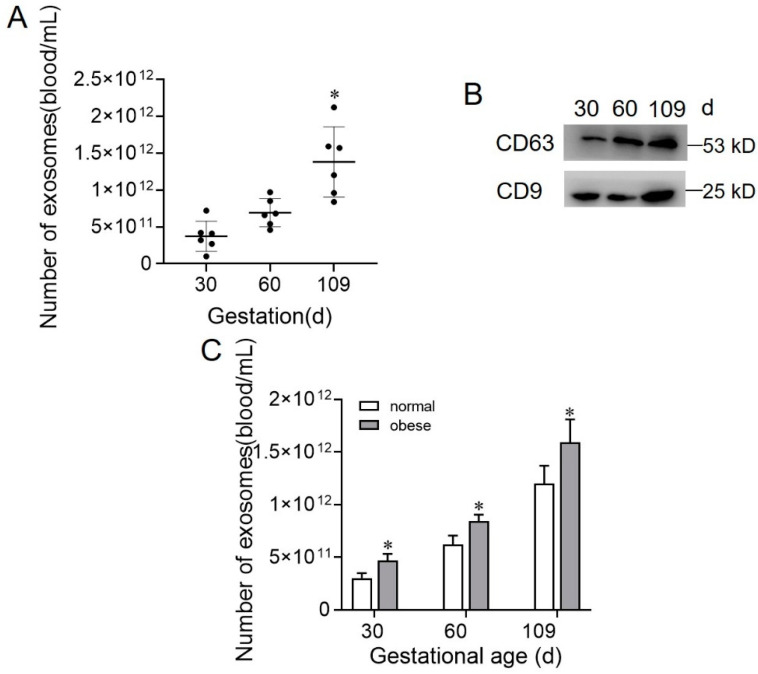
The level of plasma-derived exosomes of sows at different gestation stages and body conditions. (**A**) Total exosome number presented as the average across early (at 30 d of gestation), mid (at 60 d of gestation), and late gestation (at 109 d of gestation). (**B**) The expression of exosomal markers CD63 and CD9 at 30 d, 60 d, 90 d, and 109 d of gestation. (**C**) Total exosome number in normal (white columns) or obese (gray columns) at different gestation stages. Values are means ± SD. Values are means ± SD (n = 6). * *p* < 0.05.

To determine the effect of the gestation stage and body condition on exosome concentration during gestation, pooled exosomes were further characterized by measuring the total number of exosomes and exosomal CD63 concentration in the serial samples of sow plasma. The number of exosomes present in sow plasma progressively increased as gestation proceeded (Figure 2A). Exosomal CD63 expression levels progressively increased as gestation proceeded, while CD9 expression levels had no difference (Figure 2B). The number of exosomes was significantly higher in obese compared with normal pregnancies matched by gestational age (Figure 2C).

### 2.3. Effect of Exosome on Endothelial Cell Migration and Angiogenesis

**Figure 3:**
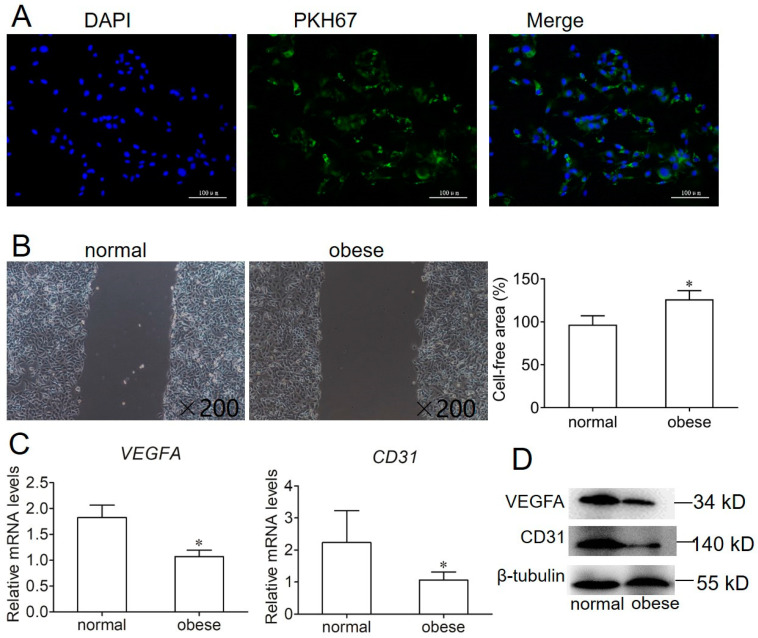
Inhibition of migration and VEGFA and CD31 levels in endothelial cells by obese-derived exosomes. (**A**) Fluorescence images of PKH67-labeled exosomes from sow were internalized into endothelial cells. (**B**) Endothelial cells were scratched and incubated with exosomes from normal or obese sows. (**C**) qPCR was performed to detect the mRNA expression of angiogenic genes *VEGFA* and *CD31* in the two groups. (**D**) Western blotting was conducted to detect the protein expression of VEGFA and CD31 in the two groups. Values are means ± SD. Values are means ± SD (n = 6). * *p* < 0.05.

To examine exosomes isolated from sow plasma that were ingested into endothelial cells, we labeled plasma-derived exosomes with the fluorescent dye PKH67. After 12 h, fluorescence microscopy analysis observed that the PKH67 label had been taken up and was transferred to the cytoplasm of endothelial cells (Figure 3A). Then, the effect of exosomes isolated from sow plasma during gestation and obtained from normal and obese sows on endothelial cell migration was determined using a scratch wound migration assay. Migrated cells (the ratio of cells that migrated to the middle) were reduced for endothelial cells with exosomes derived from obese sows (Figure 3B). Furthermore, we checked the expression of angiogenic genes, including vascular endothelial growth factor A(VEGFA) and CD31, by qPCR and Western blotting. These results showed that the mRNA and protein expression of VEGFA and CD31 significantly reduced the use of exosomes isolated from obese sow (Figure 3C,D).

### 2.4. Obese Sow Exosomal Mir-221 Is Upregulated and Impairs Angiogenesis

**Figure 4:**
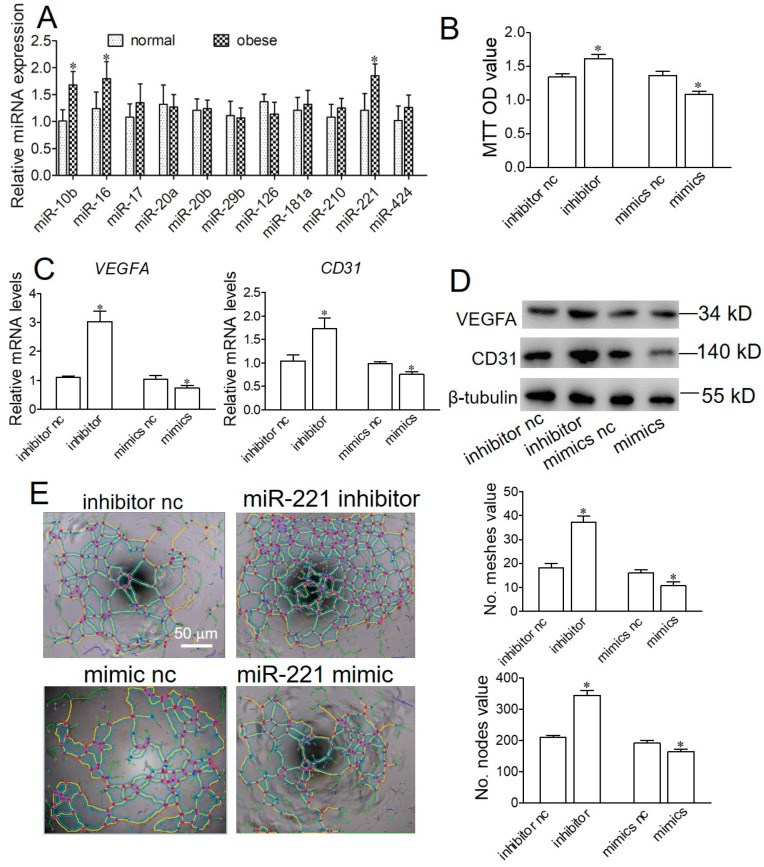
Obese sow exosomal miR 221 is secreted and impairs angiogenesis. (**A**) The expression levels of angiogenic miRNAs in plasma from normal (white columns) and obese (gray columns) sows were measured by qPCR. (**B**) Cell proliferation was examined using MTT after transfection with miR-221 inhibitor or miR-221 mimic. (**C**) qPCR was performed to detect the mRNA expression of *VEGFA* and *CD31*. (**D**) Western blotting was conducted to detect the protein expression of VEGFA and CD31. (**E**) Images of tube formation were examined in endothelial cells. Values are means ± SD (n = 6 preparative replicates). * *p* < 0.05.

To identify the differentially expressed exosomal miRNAs associated with the angiogenesis and development of the placenta, 11 miRNAs were determined. The relative expression level of exosomal miR-10b, miR-17, and miR-221 significantly increased from obese sows (Figure 4A). To elucidate the role of exosomal miRNAs in angiogenesis, we transfected endothelial cells with inhibitor-C or miRNA inhibitor, and mimic-C or miRNA mimic, and the proliferation, formation of the tubule, and VEGFA and CD31 expression were examined. The results showed that, compared with the inhibitor-C group, the proliferation, VEGFA and CD31, mRNA, and protein expression, and the number of meshes and nodes were significantly increased in the miR-221 inhibitor group (Figure 4B–E). Conversely, compared with the mimic-C group, the proliferation, VEGFA and CD31, mRNA, and protein expression, and the number of meshes and nodes were significantly increased in the miR-221 mimic group. In miR-10b and miR-17, there was no significant difference in proliferation, and angiogenesis of endothelial cells (Figure S1). These results show that exosomal miR-221 could repress the proliferation and angiogenesis of endothelial cells.

### 2.5. miR-221 Impairs Angiogenesis in Endothelial Cells by Targeting Angptl2

**Figure 5:**
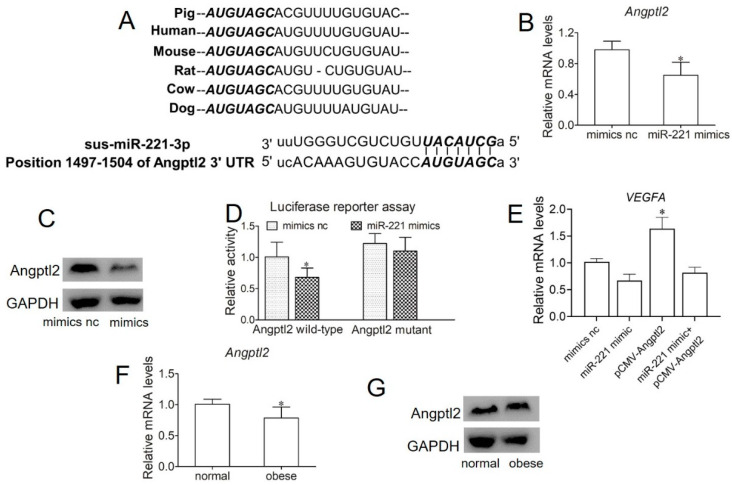
miR-221 impairs angiogenesis in endothelial cells by targeting *Angptl2*. (**A**) The sequences of sus-miR-221-3p and its predicted binding sites position of *Angptl2* 3′UTR using miRbase, miRanda, and TargetScan. (**B**) qPCR was performed to detect the mRNA expression of *Angptl2* after transfection with miR-221 inhibitor or miR-221 mimic. (**C**) Western blotting was performed to detect the protein expression of *Angptl2* after transfection with miR-221 inhibitor or miR-221 mimic. (**D**) A dual-luciferase reporter assay was performed in 293T cells. (**E**) *Angptl2* overexpression attenuated the suppressive function of miR-221-mediated angiogenesis in endothelial cells. (**F**) qPCR was performed to detect the mRNA expression of *Angptl2* after incubation with exosomes from normal or obese sows. (**G**) Western blotting was performed to detect the protein expression of *Angptl2* after incubation with exosomes from normal or obese sows. Values are means ± SD (n = 6 preparative replicates). * p < 0.05.

Angiopoietin-like protein 2 (*Angptl2*), a member of the angiopoietin-like family, is a potent facilitator of angiogenesis and promotes sprouting, migration, and proliferation in endothelial cells. We assessed the potential for *Angptl2* to be a target gene of miR-221 by bioinformatic prediction (Figure 5A). To further test the effects of miR-221 on *Angptl2*, SUVECs were treated with miR-221 mimic. The results showed that *Angptl2* was repressed by miR-221 mimic treatment at both the mRNA and protein levels (Figure 5B,C). Furthermore, to further validate whether *Angptl2* was a target gene of miR-221, we performed a dual-luciferase reporter assay to demonstrate that *Angptl2* could be directly inhibited by miR-221. The results of the luciferase assay test showed that miR-221 significantly decreased luciferase reporter activities by transfecting with wild-type *Angptl2* 3′UTR, while no effect was found by transfecting with the mutant *Angptl2* 3′UTR (Figure 5D). These results confirm that *Angptl2* is indeed a direct target of miR-221. A subsequent rescue experiment demonstrated that the overexpression of *Angptl2* may reverse the angiogenesis-mediated effect induced by miR-221(Figure 5E). Finally, to examine the effects of obese-derived exosomes on *Angptl2* expression, SUVECs were treated with exosomes derived from normal and obese sows. The results showed that *Angptl2* was reduced with obese-derived exosome treatment at both the mRNA and protein levels (Figure 5F,G). These data indicate that obese-derived exosome miR-221 could directly target the 3′UTR of *Angptl2* to regulate angiogenesis.

## 3. Discussion

In the present study, we investigated whether exosomes derived from adipose tissue induce the angiogenesis of endothelial cells associated with exosome-mediated miRNAs transfer. Our data indicate that the concentration of exosomes in sow plasma increased during pregnancy and resulted in higher levels in obese sows. Obese sow adipocyte-derived exosomes inhibited the angiogenesis of endothelial cells, and the main regulatory mechanism is enriched-miR-221 in exosomes from obese sows, which inhibited angiogenesis through targeting Angpl2. Our results suggest that adipose tissue exosome-derived miR-221 plays an important role in the angiogenesis of endothelial cells by regulating *Angpl2*.

In this study, we used a well-established and validated method of ultracentrifugation and ultrafiltration to obtain an enriched exosome fraction [17]. Exosomes displayed cup-shape characteristics and a diameter of 40–120 nm detected by electromicroscopy, Nanoparticle tracking analysis, and the measurement of the exosome markers, CD63 and CD9. These data are characteristic of exosomes and consistent with previously published data [17,18]. Our study has shown that the number and protein concentration of exosomes circulating in sow plasma increased as pregnancy progressed. Interestingly, the number and protein concentration of exosomes were significantly higher in obese sows compared with normal sow pregnancies matched by gestational age. These data are consistent with previous studies in gestational GDM and obesity [17,19]. Furthermore, obese sow adipocyte-derived exosomes inhibited endothelial cell migration and the expression of VEGFA and CD31, suggesting that the difference of exosome components may be an important factor affecting the angiogenesis of endothelial cells.

Adipose tissue is being recognized as an important endocrine organ that produces a variety of secreted factors, such as adipokines and proinflammatory cytokines [20]. Moreover, recent research has suggested the important roles of exosomes, which are secreted from adipocytes and other cells in adipose tissue [21]. Studies have shown that exosomes contain non-coding RNAs such as miRNAs. miRNAs that transfer between living cells that are involved in the interorgan communication between adipose tissue and other tissues [22]. Adipose tissue is a major source of circulating exosomal miRNAs and different adipose depots contribute different exosomal miRNAs to the circulation [13]. Several miRNAs have been identified from adipose-derived exosomes as potential biomarkers of metabolic disorders including obesity. Ying et al. (2017) reported that exosomal miR-155 derived from adipose tissue regulates whole-body glucose metabolism by modulating adipocyte functions [11]. Dang et al. (2019) reported that exosomes released from obesity adipose tissue inhibit hepatocytes’ insulin sensitivity and glucose uptake through the transfer of less miR-141-3p [16]. Pan et al. (2019) demonstrated that adipocyte-secreted exosomal miR-34a inhibits M2 macrophage polarization to promote obesity-induced adipose inflammation [23]. Furthermore, adipose tissue-derived exosomes play a pivotal role in gestational GDM and obesity [24]. Guanzon et al. (2018) identified a range of exosomal miRNAs which change expression across gestation for GDM and normal pregnancies [25]. These data suggest that the miRNA influence of adipose tissue-derived exosomes in maternal blood might act as regulators of the progression of pregnancy.

Several different adipose tissue-derived exosomal miRNAs have been reported as differentially expressed in normal, obese, or type 2 diabetic individuals [24,25]. However, specific differentially expressed miRNAs were not consistent in these papers and different patterns were reported. miR-221 was highly expressed in obese sows, and its expression level was closely related to the expression level of angiogenesis [26,27,28]. However, the effects of miR-221 on angiogenesis are still controversial. Nicoli et al. (2012) demonstrated an important regulatory node through which tip cell migration and proliferation are controlled during angiogenesis in zebrafish [26]. Chiu et al.’s (2014) study showed that miR-221 inhibits neovasculogenesis in human endothelial progenitor cells through the suppression of c-kit protein and PI3-K/Akt/eNOS signaling pathways [27]. In addition, downregulation of the miR-221/222 cluster diminished the invasion, migration, proliferation, and angiogenesis in glioblastoma [28]. In the study, the silencing of miR-221 increased the proliferation, VEGFA and CD31, mRNA, and protein expression, and the number of meshes and nodes, whereas the miR-221 mimic significantly increased the proliferation, VEGFA and CD31, mRNA, and protein expression, and the number of meshes and nodes. Taken together, all of the studies suggest that the role of miR-221 in angiogenesis depends on cell type, animal model, and physical state.

To search for the potential target genes by which miR-221 regulates angiogenesis, we adopted mirSVR analysis to predict possible targets of miR-221 that regulate angiogenesis. As shown in Figure 5, the predicted binding between miR-221 and the 3′-UTR of the *Angpl2* gene exhibited binding sites. The angiopoietin-like protein family of eight secreted glycoproteins was recently identified [29]. *Angptl2* is an adipose tissue-derived secretory glycoprotein [30]. *Angptl2* plays multiple important roles in metabolic syndrome, inflammatory carcinogenesis, and tumor metastasis [31]. This is evidence suggesting that in angiogenesis, *Angptl2* through the Tie2 tyrosine kinase receptor plays an important role in vascular remodeling [32]. Moreover, *Angptl2* is a positive regulator of angiogenesis and positively regulates ECFC vascular lumen formation [18]. In addition, a study demonstrated that miR-221 bound directly to the 3’-untranslated region of *Angptl2* and inhibited the expression of *Angptl2* in liver cancer cells, thus decreasing cell proliferation, clonogenicity, and migration/invasion [33]. Taken together, these findings suggest that obese-derived exosomes miR-221 inhibited angiogenesis by targeting *Angptl2*.

In conclusion, our findings provide novel insights into the understanding of the crosstalk between adipose tissue and placental tissue during gestation. The results suggest that adipocyte tissue-derived exosomal miR-221, which was enriched in exosomes from obese individuals and targeting *Angpl2*, may reflect the physiological state of obese animals. Therefore, further research on these issues will help us to understand the effect of maternal body condition on placental development, and it will enhance our understanding of the relationship between maternal obesity and related metabolic diseases.

## 4. Materials and Methods

### 4.1. Animal Group and Samples

The database used in this study was obtained from the research farm of Dekon Group, Sichuan Province, China. Multiparous Landrance × Large white sows (parity from 3 to 5) were collected. These were divided into two groups according to their backfat thickness at gestation. Sows with backfat from 18 to 20 mm were defined as having normal backfat (n = 15, normal), and those with backfat over 23 mm were deemed as having high backfat (n = 15, obese). All sows were fed 2.5 to 3.0 kg of a common corn-soybean-based meal gestating diet (9.4 MJ NE/kg, 14% CP and 0.6% lysine) per day, twice daily at 07:00 and 16:00. All sows were individually housed and provided water freely. Serial blood samples (5–7 mL) (BD Vacutainer PLUS Tubes, EDTA) at 30 (early), 60 (mid), and 109 (late) days were collected. Blood samples were transferred into chilled nonheparinized vacutainer tubes. Plasma samples were stored at −80 °C for subsequent assay. 

### 4.2. Isolation of Exosomes from Plasma Samples

The extraction of exosomes in plasma was performed as previously described [17]. Briefly, plasma was diluted with an equal volume of PBS (pH 7.4) and centrifuged at 2000× *g* for 30 min at 4 °C. The supernatant was then centrifuged at 12,000× *g* and 4 °C for 45 min. The resultant supernatant fluid (2 mL) was transferred to an ultracentrifuge tube (10 mL, Beckman, CA, USA) and centrifuged at 100,000× *g* for 2 h (T-8100, fixed angle ultracentrifuge rotor, Sorvall, Waltham, MA, USA). The pellet was suspended in PBS (10 mL) and filtered through a 0.22 μm filter (Steritop; Millipore, Billerica, MA, USA) and then centrifuged at 100,000× *g* for 2 h. The 100,000× *g* pellet was resuspended in 500 μL PBS and stored −80 °C until exosome purification

### 4.3. Exosome Characterization

Exosomes were characterized by nanoparticle tracking analysis (NTA), transmission electron microscopy (TEM), and Western blot. The sizes of the exosomes were determined using a Zetasizer Nano ZSP (Malvern Instruments, Malvern, UK). Pellets of extracellular vesicles were resuspended in 1 mL of 0.2 μm-filtered PBS using a 1 mL pipette. Samples were manually injected into the sample chamber at an ambient temperature. The data were analyzed using the NTA analytical software (version 2.3).

Exosome morphology was determined using a transmission electron microscopy (JEOL Ltd., Tokyo, Japan). the exosome pellet was placed in a droplet of 2% glutaraldehyde in PBS buffer at pH 7.2 and fixed overnight at 4 °C. After thoroughly mixing, transfer a 20 μL sample onto parafilm with a pipette. Lightly cover this droplet with a stencil coated copper mesh and coat the surface for 2 min at room temperature. Carefully strip off the excess solution with hardened ash-free filter paper and transfer the copper mesh to 2% uranyl acetate drops and store for 2 min at room temperature. Samples were stored side up in a copper-coated grid at room temperature and viewed through a transmission electron microscope for 2 h. Western blotting was used to examine the specific exosome surface markers, CD63 and CD9, which could also be expressed in exosomes. The concentrations of exosomes in sow circulation were quantified using a CD63 ELISA as previously described [34]. To monitor exosome trafficking, exosomes were labeled with PKH67 fluorescent dye using the PKH67 fluorescent cell linker kit (Sigma-Aldrich). Exosomes were fluorescently labeled using the PKH67 dye according to the manufacturer’s protocol.

### 4.4. Endothelial Cell Culture

Swine umbilicus veins endothelial cells (SUVECs) were used to determine the bioactivity of exosomes isolated from maternal plasma. SUVECs primary cultures (37 °C, 5% CO_2_) were isolated by enzymatic digestion using Collagenase Type II (Gibco Life Technologies, Carlsbad, CA, USA) as previously described [35]. Cells were cultured in a primary culture medium containing 2% exosome-depleted FBS (culture medium was depleted of the contaminating exosomes using the same protocol for exosome isolation described previously [36]), and the exosome-free culture medium was confirmed by electron microscopy) for 24 h before experiments.

### 4.5. Endothelial Cell Migration

We performed a wound-healing assay as described previously [37]. The cells were incubated in a 24-well plate for 6 h and wounded in a line across the well using a 100 μL pipette tip. The loose cells were removed by washing with PBS, and a 500 μL test medium (normal/obese exosomes) was added, followed by incubation at 37 °C. Images were recorded at 24 h. The wound area was determined using a fluorescence inversion microscope system (Nikon, Tokyo, Japan).

### 4.6. Cell Proliferation Assay

SUVECs were suspended in a culture medium and seeded in a 12-well plate at 1 × 10^6^ cells per well. After 24 h, the cells were cultured with FBS-free medium for 6 h. Then, the SUVECs were transfected with miR-221 inhibitor or mimics for 48 h. Cell proliferation was detected by MTT Assay Kit (Beyotime Institute of Biotechnology Shanghai, Shanghai, China), following the manufacturer’s instructions.

### 4.7. miRNA and Plasmid Transfection

SUVECs were seeded in cell culture plates and cultured for 24 h. Lipofectamine 2000 (Thermo Fisher, Waltham, MA, USA) with miRNA inhibitor or mimic (Suzhou GenePharma, Suzhou, China) was added into cells and incubated at 37 °C for 48 h. The miRNA inhibitor, mimic, and plasmid were transfected according to the manufacturer’s instructions. Subsequently, RNA and protein were extracted to determine the changes. For the rescue experiment, The *Angptl2* plasmid was obtained from Suzhou Genechem Co., Ltd. (Suzhou, China). The experiment included four groups: mimics nc, miR-211 mimics, *Angptl2* plasmid, and miR-221 mimics + *Angptl2* plasmid. Each group of mimics and *Angptl2* were co-transfected into SUVECs. The cells were harvested 24 h after transfection.

### 4.8. Luciferase Reporter Assay

The *Angptl2* 3′UTR region was amplified and cloned into a firefly luciferase reporter vector (FLuc-*Angptl2*-wild type). The position site 1497-1504 (5′ AUGUAGCA 3′) of 3′UTR of *Angptl2* was mutated to 5′ AUCAUCCA 3′ and synthesized by GenePharma of China. Then, a mutant version of the *Angptl2* 3′UTR reporter plasmid (FLuc-*Angptl2*-mutant) was constructed. The 293T cells were co-transfected with the reporter plasmid and miRNA nc or miRNA mimic plus the phRL-TK vector (Promega, Madison, WI, USA) using Lipofectamine 2000 reagent (Life Technologies, Carlsbad, CA, USA). Luciferase activity was determined using a Dual-Glo luciferase assay system (Promega, Madison, WI, USA) at 48 h according to the manufacturer’s instructions.

### 4.9. Tube Formation Assay

Tube formation was examined as reported previously with some modification [38]. Matrigel Basement Membrane Matrix (Corning) was warmed up at room temperature and 10 μL of basement matrix was placed directly into the center of each well of a 24-well plate. The plate was placed at 37 °C for 30 min to allow the gel to set. Then, 5 × 10^4^ SUVECs were added in 500 µL of media and incubated for 24 h. Phase-contrast images (5 images/well) were acquired using a Nikon TMS inverted microscope (×10 magnification), and the mean number of tubes and nodes of the tubular structures was counted manually using ImageJ software to form each n-number from each donor.

### 4.10. RNA Extraction, Reverse Transcriptase (RT)-PCR, and Real-Time RT-PCR

Total RNA from exosomes and cultured cells was extracted using the TRIzol reagent (Invitrogen, Carlsbad, CA, USA) according to the manufacturer’s instructions. The ReverTra Ace qPCR RT Kit (TOYOBO, Japan) was used for quantitative miRNA. A reverse transcription reaction (20 μL) was performed consisting of a mixture of 1 μg purified total RNA, and 2 μL primers (10 mM) of stem-loop RT (miR-221: U6, 1:1), 2.5 μL of 5 × RT buffer, 2 μL of dNTPs (10 mM), 1 μL of ReverTra Ace (100 U/μL), 0.5 μL of RNase inhibitor (40 U/μL), and 20 μL of DEPC-H2O. The reaction mixture was incubated at 42 °C for 60 min, 95 °C for 5 min, and then held at 4 °C. U6 served as an endogenous control [39]. For mRNA quantification, PCR primers were synthesized from Sangon Biotech (Shanghai, China) (Table S1). Reverse transcription and RT-PCR were performed as above. *β-actin* was used as the internal mRNA reference. Quantitative real-time PCR was performed using iQ SYBR Green Supermix (BioRad, Hercules, CA, USA) on a CFXTM 384 Touch qPCR system (BioRad, Hercules, CA, USA). The relative expression level was estimated by calculating 2^−^^ΔΔCT^.

### 4.11. Western Blotting

Protein level determination was performed as described previously [40]. Cells were trypsinized and pelleted by centrifugation at 1000× *g* for 5 min. Then, protein lysates were extracted with a solution containing 20 mM Tris-HCl (pH 7.5), 150 mM NaCl, 1% Triton X-100, 10 mM Na_4_P_2_O_7_, 1 mM Na_3_VO_4_, 2 mM EDTA, 0.5 mM leupeptin, and 1 mM PMSF (Beyotime, Shanghai, China). A BCA protein assay kit (Beyotime, Shanghai, China) was used to determine the protein concentrations, and proteins were separated by 10% SDS-PAGE. After proteins were transferred onto a polyvinylidene difluoride (PVDF) membrane (Bio-Rad, Hercules, CA, USA) for protein separation. Then, the blots were blocked in TBST buffer (10 mM Tris-HCl (pH 7.6), 150 mM NaCl, 0.1% Tween) containing 5% skim milk powder supplemented with blocking agent for 1 h. After blocking, the membrane was incubated with primary antibodies against CD63 (no. 134045, Abcam), CD9 (no. 13403, Cell Signaling Technology Inc., Danvers, MA, USA), VEGFA (no. A12303, ABclonal, Wuhan, China), CD31 (no. A0378, ABclonal, Wuhan, China), Angptl2 (no. DF12557, Affinity, China), β-tubulin (no. 2128, Cell Signaling Technology Inc., Danvers, MA, USA), and GAPDH (no. 5174, Cell Signaling Technology Inc., Danvers, MA, USA) overnight at 4 °C. Anti-mouse or anti-rabbit IgG-HRP (Invitrogen, Carlsbad, CA, USA) were used as secondary detection antibodies for 1 h at room temperature. Finally, immunoreactive bands were detected using an enhanced chemiluminescence detection kit (Thermo Fisher Scientific, Waltham, MA, USA) according to the manufacturer’s instructions. Protein levels were normalized to β-tubulin or GAPDH using Image J analysis software.

### 4.12. Statistical Analysis

Data are expressed as mean ± SD. One-way analysis of variance (ANOVA) tests were conducted, along with corresponding posttests, as indicated. Calculation of the average was performed using at least three biological replicas. *p* < 0.05 was considered significant. Statistical analyses were carried out using GraphPad Prism version 8 for Windows.

## Data Availability

The data presented in this study are available within the article text, figures and supplementary materials.

## Supplementary Materials

The following are available online at https://www.mdpi.com/article/10.3390/ijms221910343/s1.

## Abbreviations

Angpl2	Angiopoietin-like protein 2
IUGR	Intrauterine growth restriction
LBW	Low birth weight
LGA	Large for gestational age
miRNAs	microRNAs
NTA	Nanoparticle tracking analysis
PPARγ	Peroxisome proliferator-activated receptor γ
SGA	Small for gestational age
SUVECs	Swine umbilicus veins endothelial cells
TEM	Transmission electron microscopy
VEGFA	Vascular endothelial growth factor A

## References

[1] Stang J., Huffman L.G. (2016). Position of the academy of nutrition and dietetics: Obesity, reproduction, and pregnancy outcomes. J. Acda. Nutr. Diet..

[2] Higgins L., Greenwood S.L., Wareing M., Sibley C.P., Mills T.A. (2011). Obesity and the placenta: A consideration of nutrient exchange mechanisms in relation to aberrant fetal growth. Placenta.

[3] Zhou Y., Xu T., Cai A., Wu Y., Wei H., Jiang S., Peng J. (2018). Excessive backfat of sows at 109 d of gestation induces lipotoxic placental environment and is associated with declining reproductive performance. J. Anim. Sci..

[4] Kim J.S., Yang X., Pangeni D., Baidoo S.K. (2015). Relationship between backfat thickness of sows during late gestation and reproductive efficiency at different parities. ACTA Agric. Scand. A—Anim..

[5] Leddy M.A., Power M.L., Schulkin J. (2008). The impact of maternal obesity on maternal and fetal health. Rev. Obstet. Gynecol..

[6] Weckman A.M., Ngai M., Wright J., McDonald C.R., Kain K.C. (2019). The impact of infection in pregnancy on placental vascular development and adverse birth outcomes. Front. Microbiol..

[7] Jarvie E., Hauguel-de-Mouzon S., Nelson S.M., Sattar N., Catalano P.M., Freeman D.J. (2010). Lipotoxicity in obese pregnancy and its potential role in adverse pregnancy outcome and obesity in the offspring. Clin. Sci..

[8] Saben J., Lindsey F., Zhong Y., Thakali K., Badger T.M., Andres A., Gomez-Acevedo H., Shankar K. (2014). Maternal obesity is associated with a lipotoxic placental environment. Placenta.

[9] Xu Y., Lai Y., Cao L., Li Y., Chen G., Chen L. (2020). Human umbilical cord mesenchymal stem cells-derived exosomal microRNA-451a represses epithelial–mesenchymal transition of hepatocellular carcinoma cells by inhibiting ADAM10. RNA. Biol..

[10] Ahima R.S., Flier J.S. (2000). Adipose tissue as an endocrine organ. Trends Endocrin. Met..

[11] Ying W., Riopel M., Bandyopadhyay G., Dong Y., Birmingham A., Seo J.B., Ofrecio J.M., Wollam J., Hernandez-Carretero A., Fu W. (2017). Adipose tissue macrophage-derived exosomal miRNAs can modulate in vivo and in vitro insulin sensitivity. Cell.

[12] Crewe C., Joffin N., Rutkowski J.M., Kim M., Zhang F., Towler D.A., Gordillo R., Scherer P.E. (2018). An endothelial-to-adipocyte extracellular vesicle axis governed by metabolic state. Cell.

[13] Thomou T., Mori M.A., Dreyfuss J.M., Konishi M., Sakaguchi M., Wolfrum C., Rao T.N., Winnay J.N., Garcia-Martin R., Grinspoon S.K. (2017). Adipose-derived circulating miRNAs regulate gene expression in other tissues. Nature.

[14] Yu Y., Du H., Wei S., Feng L., Li J., Yao F., Zhang M., Hatch G.M., Chen L. (2018). Adipocyte-derived exosomal MiR-27a induces insulin resistance in skeletal muscle through repression of PPARγ. Theranostics.

[15] Richardson M.R., Robbins E.P., Vemula S., Critser P.J., Whittington C., Voytik-Harbin S.L., Yoder M.C. (2014). Angiopoietin-like protein 2 regulates endothelial colony forming cell vasculogenesis. Angiogenesis.

[16] Dang S.Y., Leng Y., Wang Z.X., Xiao X., Zhang X., Wen T., Gong H.Z., Hong A., Ma Y. (2019). Exosomal transfer of obesity adipose tissue for decreased miR-141-3p mediate insulin resistance of hepatocytes. Int. J. Biol. Sci..

[17] Sharma P., Ludwig S., Muller L., Hong C.S., Kirkwood J.M., Ferrone S., Whiteside T.L. (2018). Immunoaffinity-based isolation of melanoma cell-derived exosomes from plasma of patients with melanoma. J. Extracell. Vesicles.

[18] Salomon C., Scholz-Romero K., Sarker S., Sweeney E., Kobayashi M., Correa P., Longo S., Duncombe G., Mitchell M.D., Rice G.E. (2016). Gestational diabetes mellitus is associated with changes in the concentration and bioactivity of placenta-derived exosomes in maternal circulation across gestation. Diabetes.

[19] Barranco I., Padilla L., Parrilla I., Álvarez-Barrientos A., Pérez-Patiño C., Peña F.J., Martínez E.A., Rodriguez-Martínez H., Roca J. (2019). Extracellular vesicles isolated from porcine seminal plasma exhibit different tetraspanin expression profiles. Sci. Rep..

[20] Jayabalan N., Nair S., Nuzhat Z., Rice G.E., Zuñiga F.A., Sobrevia L., Leiva A., Sanhueza C., Gutiérrez J.A., Lappas M. (2017). Cross Talk between Adipose Tissue and Placenta in Obese and Gestational Diabetes Mellitus Pregnancies via Exosomes. Front. Endocrinol..

[21] Lee M.W., Lee M., Oh K.J. (2019). Adipose tissue-derived signatures for obesity and type 2 diabetes: Adipokines, batokines and microRNAs. J. Clin. Med..

[22] Kita S., Maeda N., Shimomura I. (2019). Interorgan communication by exosomes, adipose tissue, and adiponectin in metabolic syndrome. J. Clin. Invest..

[23] Pan Y., Hui X., Hoo R.L.C., Ye D., Chan C.Y.C., Feng T., Wang Y., Lam K.S.L., Xu A. (2019). Adipocyte-secreted exosomal microRNA-34a inhibits M2 macrophage polarization to promote obesity-induced adipose inflammation. J. Clin. Invest..

[24] Otero-Ortega L., Laso-García F., Gómez-de Frutos M., Fuentes B., Diekhorst L., Díez-Tejedor E., Gutiérrez-Fernández M. (2019). Role of exosomes as a treatment and potential biomarker for stroke. Transl. Stroke Res..

[25] Guanzon D., Lai A., Scholz-Romero K., Zuniga F., Diaz E., McIntyre H.D., Lappas M., Salomón C. (2018). Circulating Exosomal miRNA Signature in Pregnancies with Gestational Diabetes Mellitus across Gestation. Diabetes.

[26] Nicoli S., Knyphausen C.P., Zhu L.J., Lakshmanan A., Lawson N.D. (2012). miR-221 is required for endothelial tip cell behaviors during vascular development. Dev. Cell.

[27] Chiu S.C., Chiang E.P., Tsai S.Y., Wang F.Y., Pai M.H., Syu J.N., Cheng C.C., Rodriguez R.L., Tang F.Y. (2014). Eicosapentaenoic acid induces neovasculogenesis in human endothelial progenitor cells by modulating c-kit protein and PI3-K/Akt/eNOS signaling pathways. J. Nutr. Biochem..

[28] Wu X.G., Zhou C.F., Zhang Y.M., Yan R.M., Wei W.F., Chen X.J., Yi H.Y., Liang L.J., Fan L.S., Liang L. (2019). Cancer-derived exosomal miR-221-3p promotes angiogenesis by targeting THBS2 in cervical squamous cell carcinoma. Angiogenesis.

[29] Ohno T., Yamamoto G., Hayashi J.I., Nishida E., Goto H., Sasaki Y., Kikuchi T., Fukuda M., Hasegawa Y., Mogi M. (2017). Angiopoietin-like protein 2 regulates Porphyromonas gingivalis lipopolysaccharide-induced inflammatory response in human gingival epithelial cells. PLoS ONE.

[30] Kadomatsu T., Endo M., Miyata K., Oike Y. (2014). Diverse roles of ANGPTL2 in physiology and pathophysiology. Trends. Endocrinol. Metab..

[31] Wang X., Hu Z., Wang Z., Cui Y., Cui X. (2019). Angiopoietin-like protein 2 is an important facilitator of tumor proliferation, metastasis, angiogenesis and glycolysis in osteosarcoma. Am. J. Transl. Res..

[32] Oike Y., Urano T., Ito Y., Fukuhara S., Akao M., Mochizuki N. (2006). Angiopoietin-Like Protein 2 Induces Angiogenesis, Lymphangiogenesis, and Leukocytes Recruitment, Resulting in Enhancement of Inflammation. Circulation.

[33] He X.X., Guo A.Y., Xu C.R., Chang Y., Xiang G.Y., Gong J., Dan Z.L., Tian D.A., Liao J.Z., Lin J.S. (2014). Bioinformatics analysis identifies miR-221 as a core regulator in hepatocellular carcinoma and its silencing suppresses tumor properties. Oncol. Rep..

[34] Salomon C., Torres M.J., Kobayashi M., Scholz-Romero K., Sobrevia L., Dobierzewska A., Illanes S.E., Mitchell M.D., Rice G.E. (2014). A gestational profile of placental exosomes in maternal plasma and their effects on endothelial cell migration. PLoS ONE.

[35] Ning P., Zhang Y., Guo K., Chen R., Liang W., Lin Z., Li H. (2014). Discovering up-regulated VEGF-C expression in swine umbilical vein endothelial cells by classical swine fever virus Shimen. Vet. Res..

[36] Lee Y.R., Kim G., Tak W.Y., Jang S.Y., Kweon Y.O., Park J.G., Lee H.W., Han Y.S., Chun J.M., Park S.Y. (2019). Circulating exosomal noncoding RNAs as prognostic biomarkers in human hepatocellular carcinoma. Int. J. Cancer.

[37] Hwang S.H., Lee B.H., Choi S.H., Kim H.J., Won K.J., Lee H.M., Rhim H., Kim H.C., Nah S.Y. (2016). Effects of gintonin on the proliferation, migration, and tube formation of human umbilical-vein endothelial cells: Involvement of lysophosphatidic-acid receptors and vascular-endothelial-growth-factor signaling. J. Ginseng Res..

[38] DeCicco-Skinner K.L., Henry G.H., Cataisson C., Tabib T., Gwilliam J.C., Watson N.J., Bullwinkle E.M., Falkenburg L., O’Neill R.C., Morin A. (2014). Endothelial cell tube formation assay for the in vitro study of angiogenesis. J. Vis. Exp..

[39] Peng J., Zhou Y., Deng Z., Zhang H., Wu Y., Song T., Yang Y., Wei H., Peng J. (2018). miR-221 negatively regulates inflammation and insulin sensitivity in white adipose tissue by repression of sirtuin-1 (SIRT1). J. Cell. Biochem..

[40] Zhou Y.F., Ren J., Song T.X., Peng J., Wei H.K. (2016). Methionine Regulates mTORC1 via the T1R1/T1R3-PLCβ-Ca^2+^-ERK1/2 Signal Transduction Process in C2C12 Cells. Int. J. Mol. Sci..

