# Supplementary material for: Circulating Exosomal miR-221 from Maternal Obesity Inhibits Angiogenesis via Targeting Angptl2
Source: Int J Mol Sci. 2021 Sep 26;22(19):10343. doi: 10.3390/ijms221910343 (PMC8508603; doi:10.3390/ijms221910343)
Supplement: Supplementary file 1 [file ijms-22-10343-s001.zip › ijms-1371319-supplementary.pdf]

Supplementary data

Table S1. Primer sets used for real-time quantitative PCR.

Gene Name	Accession No.	Sequence (sense/antisense 5′-3′)	Product Size
Angptl2	NM_001109946.1	TTCGGTATTCTCGGTCCCTG<br>GCCCACACACCGTGTCTATT	159bp
CD31	NM_213907.1	AAGGAAGTGACCTTCTGGCG<br>ACCTCCTCGCTCAGGAGAAT	202bp
VEGFA	NM_214084.1	ATGGCAGAAGGAGACCAGAA<br>ATGGCGATGTTGAACTCCTC	224bp
β-actin	DQ452569.1	CCAGGTCATCACCATCGG<br>CCGTGTTGGCGTAGAGGT	158bp

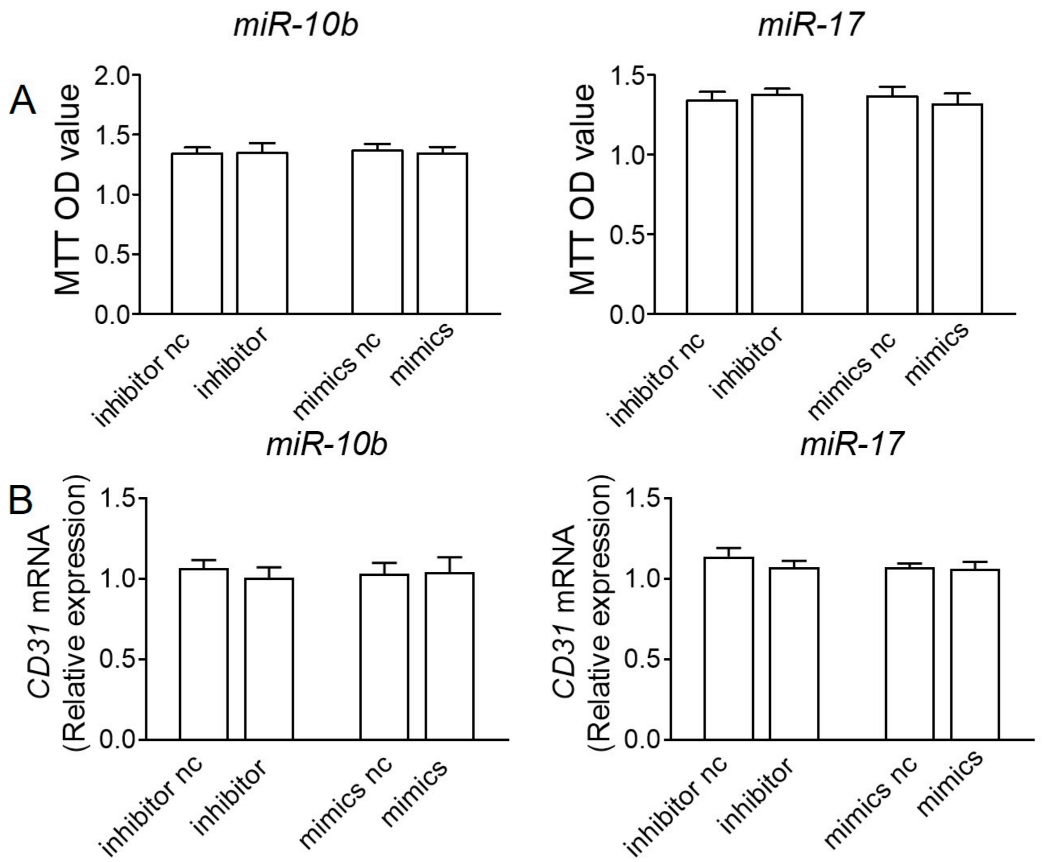

Figure S1. The effects of miR-10b and miR-17 for endothelial cells proliferation and mRNA of *CD31.* (**A**) Cell proliferation was examined using MTT after transfection with miR-10b or miR-17 inhibitor or mimic. (**B**) qPCR was performed to detect the mRNA expression of *CD31*.
